# A case of severe pulmonary embolism after total robotic hysterectomy despite venous thromboembolism prophylaxis as prescribed

**DOI:** 10.1016/j.ijscr.2024.109396

**Published:** 2024-02-15

**Authors:** Shohei Tanabe, Kotaro Ichida, Kiyoshi Niiya, Syuji Morishima

**Affiliations:** Kobe City Medical Center, West Hospital, Japan

**Keywords:** Venous thromboembolism, Prophylaxis, Robotic surgery, Total hysterectomy, Case report

## Abstract

**Introduction:**

We report the case of a patient who developed a severe pulmonary embolus postoperatively despite perioperative venous thromboembolism (VTE) prophylaxis as prescribed.

**Presentation of case:**

A 50-year-old female patient underwent a robotic total hysterectomy for uterine fibroids. Her perioperative VTE risk was assessed as moderate, and compression and intermittent air compression stockings were used postoperatively until the morning following the surgery. The surgery was uneventful, and the patient was discharged on postoperative day 4. On postoperative day 19, the patient experienced rapid dyspnea and was diagnosed with a severe pulmonary embolus.

**Discussion:**

Heparin, a tissue-type plasminogen activator, and a catecholamine were administered, and the patient recovered well.

**Conclusion:**

VTE measures in minimally invasive gynecologic surgery are not well defined, and future thrombotic risk assessments specific to minimally invasive gynecologic surgery may be necessary.

## Highlights

• We experienced a case of severe pulmonary embolism after a robotic total hysterectomy.
• The case was treated perioperatively with anti-thrombotic prophylaxis.
• The question remains whether thromboprophylaxis should be continued long after discharge from the hospital.

## 1 Introduction

Pulmonary emboli are dangerous conditions that cause acute right ventricular failure. The turnaround time for pulmonary emboli varies greatly depending on the patient's cardiopulmonary status and the size of the thrombus [1]. In gynecological surgery patients, this includes previous venous thrombosis, age, surgery type, presence of malignancy, and obesity [2]. It is important that thromboprophylaxis be used appropriately according to risk factors [3]. Past reports indicate that gynecologic surgery has a lower risk of pulmonary embolism than that of other surgeries [4]. However, the risk of pulmonary embolization after robotic surgery has not been fully investigated [5].

In this report, we describe the case of a patient who underwent a robotic total hysterectomy and developed a severe pulmonary embolus after surgery despite measures to prevent venous thromboembolism (VTE).

This work has been reported in line with the SCARE criteria [6].

## 2 Presentation of case

The patient was a 50-year-old female with a history of G6T4 (NVDx4) P2A0L4 and appendicitis for which she had undergone surgery. The patient was a healthy, middle-class Asian woman. There was no other medical history, family history of VTE, or smoking history. The patient had a BMI of 26.5 kg/m^2^ and was of standard build. Due to the presence of uterine fibroids and persistent genital bleeding, the decision was made to perform a total hysterectomy. The fibroids were small and of standard size. The patient requested a total hysterectomy. The patient was referred to our obstetrics and gynecology department by her primary care physician due to genital bleeding. Uterine fibroids were found on ultrasonography, and a robotic total hysterectomy was scheduled approximately 6 months after the initial visit. The operation time was 2 h 53 min, and the blood loss was 25 ml. The uterus weighed 202 g. Before surgery, the patient was assessed for deep venous thrombosis (DVT) risk based on the Guidelines for Diagnosis, Treatment and Prevention of Pulmonary Thromboembolism and Deep Vein Thrombosis (JCS, 2017), a commonly used guideline in Japan. Based on these guidelines, DVT risk was assessed before surgery as intermediate (age > 40 years and operative time > 45 min). Based on this evaluation, graded compression and intermittent pneumatic stockings were used postoperatively to prevent a VTE.

**Fig. 1:**
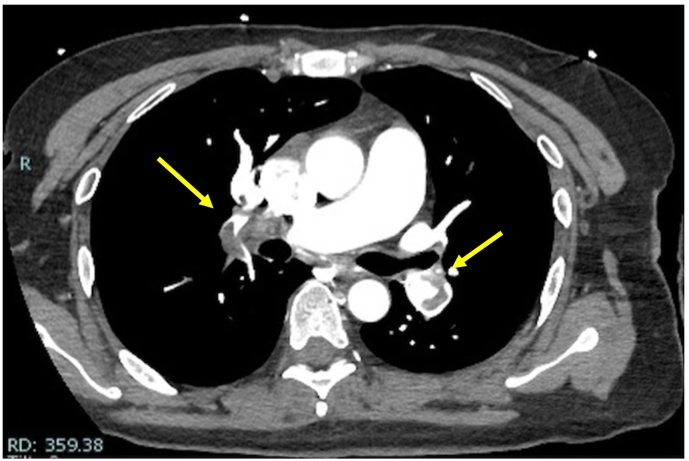
Computed tomography at admission. Yellow arrows indicate the thrombi.

**Fig. 2:**
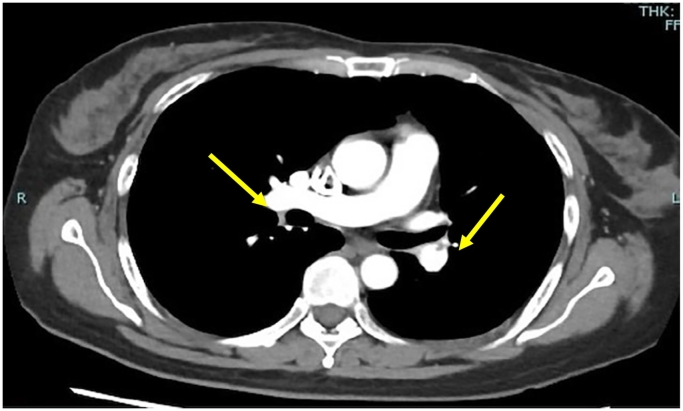
Computed tomography on the 8th day of hospitalization. The thrombi have disappeared.

The patient started walking on the first postoperative day when graded compression and intermittent pneumatic stockings were terminated. The patient's postoperative course was good, and she was discharged from the hospital on the fourth postoperative day. On the 19th day after surgery, the patient developed respiratory distress upon returning home from work. She fainted in her room, and her family called for emergency medical assistance. The patient had fainted three times before the Emergency Medical Services arrived. The patient was transported to another hospital's Emergency and critical care medical center. On arrival at the hospital, her vital signs were: blood pressure (BP): 89/68 mmHg, heart rate (HR): 128 bpm, and saturation of percutaneous oxygen (SpO_2_): 97 % (Oxygen 6 L). The patient was classified as class V, the most severe, on the pulmonary embolism severity index (PESI) score, which is used to classify the severity of pulmonary emboli. Transthoracic echocardiography showed prominent enlargement of the right ventricle, and computed tomography (CT) revealed thrombi in both pulmonary arteries (Fig. 1), leading to a diagnosis of pulmonary embolism. Blood tests showed an elevated D-dimer level of 19.4 (μg/ml) (Table 1). After diagnosis, heparin administration was initiated. A catecholamine was also used. Administration of tissue plasminogen activator (t-PA) is relatively contraindicated immediately after surgery [1]. However, because the patient had a severe pulmonary embolus, thrombolysis was expected to be beneficial. Since there was no bleeding from the surgical wound, t-PA was administered on the day after admission. On the third day of admission, her vital signs normalized, the catecholamine was discontinued, and direct oral anticoagulants (DOACs) were initiated. Rehabilitation was also initiated, and the D-dimer levels gradually decreased (Table 2). On the 8th day of hospitalization, a CT scan showed the disappearance of the large pulmonary embolus (Fig. 2), and the patient was discharged on the 9th day of hospitalization. In the outpatient clinic after discharge, she underwent tests for antinuclear antibodies, lupus anticoagulants, anticardiolipin antibodies, protein S, and protein C, all of which were negative, considering possible diseases that could cause excessive blood coagulation.

**Table 1:** Results of blood tests conducted at the time of admission.

Laboratory values		Reference range
Blood values		
White blood cell count (/μl)	10,800	3900–9800
Hemoglobin (g/dl)	12.5	11.1–15.1
d-Dimer(μg/ml)	19.4	<1.0
NT-proBNP (pg/ml)	241	<40
Troponin (ng/ml)	0.016	<0.1
CK (U/L)	31	<46
d-Dimer(μg/ml)	19.4	0.00–0.99

CK, Creatine kinase; NT-proBNP, N-terminal pro–B-type natriuretic peptide.

**Table 2:** Changes in D-dimer levels.

	Day 1	Day 2	Day 3	Day 4	Day 8
D-dimer level (μg/ml)	19.4	15.28	17.94	6.37	6.23

## 3 Discussion

The Guidelines for the Diagnosis, Treatment and Prevention of Pulmonary Thromboembolism and Deep Vein Thrombosis (JCS 2017) are based on the American College of Chest Physicians guidelines [7]. The criteria for combining mechanical prophylaxis with intermittent pneumatic compression and pharmacological prophylaxis with low-molecular-weight heparin or other agents for VTE prophylaxis in abdominopelvic surgery, depending on risk, are described. They also report that the risk of VTE and bleeding complications must be considered. The guidelines also do not recommend regular monitoring using venous compression ultrasound to detect asymptomatic leg venous thrombi, only emphasizing the importance of assessing VTE risk.

It is generally recognized that minimally invasive surgeries, such as robot-assisted surgery, have fewer perioperative complications, although there are inherent risks, such as intraoperative patient positioning, teaching training, and surgical instruments [8]. However, criteria for VTE prophylaxis in minimally invasive gynecological surgery have not yet been established. Previously, postoperative anticoagulation was recommended for patients with a history of thrombosis, advanced age, surgery for malignant tumors, or prolonged surgery [9]. However, in a study on robotic surgery for endometrial cancer, VTE itself was rare; therefore, the creation of prevention guidelines is required in the future [10]. Considering the above, there are currently no clear standards for VTE prophylaxis in robotic total hysterectomy, and whether VTE prophylaxis is necessary remains unknown.

Considering a previous report on VTEs in a group of patients who did not receive long-term postoperative thromboprophylaxis [11], it is possible that pulmonary embolism would not have occurred in this case if long-term thromboprophylaxis had been administered. However, given that urologists have reported more hemorrhagic complications in patients treated with anticoagulation after robot-assisted partial nephrectomy (RAPN) [12], a similar increase in hemorrhagic complications can be expected in patients who undergo robotic total hysterectomy in gynecology. A similar increase in bleeding complications was expected in the gynecological post-robotic hysterectomy case group. Thus, a new risk assessment tool that balances VTE prophylaxis and bleeding complications is needed.

## 4 Conclusion

Future VTE risk assessment and preventive measures specific to minimally invasive gynecological surgery cases may be needed. VTE prophylaxis measures may also need to be considered for both the perioperative period and the long term.

## Informed consent

Informed consent was obtained from all individuals included in this study. Written informed consent was obtained from the patient for publication and any accompanying images. A copy of the written consent is available for review by the Editor-in-Chief of this journal on request.

## Ethical approval

Ethical approval for this study (Ethical Committee No. 23–027) was provided by the Ethical Committee of Kobe City Medical Center West Hospital, Kobe, Japan on 13 December 2015.

## Funding

This study did not receive any specific grants from funding agencies in the public, commercial, or non-profit sectors.

## Author contribution

All coauthors reviewed the manuscript draft and revised it critically for intellectual content. All co-authors approved the final version of the manuscript for publication.

## Guarantor

Shohei Tanabe.

## Research registration number

1. Name of the registry: Research Registry
2. Unique identifying number or registration ID: researchregistry9897
3. Hyperlink to your specific registration (must be publicly accessible and will be checked): https://www.researchregistry.com/browse-the-registry#home/registrationdetails/659e12321ca40c00270559d9/.

## Declaration of competing interest

The authors state no conflicts of interest.
